# The Composition, Diversity and Predictive Metabolic Profiles of Bacteria Associated With the Gut Digesta of Five Sea Urchins in Luhuitou Fringing Reef (Northern South China Sea)

**DOI:** 10.3389/fmicb.2019.01168

**Published:** 2019-05-28

**Authors:** Qiucui Yao, Kefu Yu, Jiayuan Liang, Yinghui Wang, Baoqing Hu, Xueyong Huang, Biao Chen, Zhenjun Qin

**Affiliations:** ^1^Coral Reef Research Center of China, Guangxi University, Nanning, China; ^2^Guangxi Laboratory on the Study of Coral Reefs in the South China Sea, Guangxi University, Nanning, China; ^3^School of Marine Sciences, Guangxi University, Nanning, China; ^4^College of Forestry, Guangxi University, Nanning, China; ^5^Key Laboratory of Environment Change and Resources Use in Beibu Gulf, Ministry of Education, Guangxi Teachers Education University, Nanning, China

**Keywords:** sea urchin, gut digesta bacteria, coral reef, degradation, restoration

## Abstract

Sea urchins strongly affect reef ecology, and the bacteria associated with their gut digesta have not been well studied in coral reefs. In the current study, we analyze the bacterial composition of five sea urchin species collected from Luhuitou fringing reef, namely *Stomopneustes variolaris*, *Diadema setosum*, *Echinothrix calamaris*, *Diadema savignyi*, and *Tripneustes gratilla*, using high-throughput 16S rRNA gene-based pyrosequencing. *Propionigenium*, *Prolixibacter*, and *Photobacterium* were found to be the dominant bacterial genera in all five species. Interestingly, four sea urchin species, including *S. variolaris*, *D. setosum*, *E. calamaris*, and *D. savignyi*, displayed a higher mean total abundance of the three bacterial genera (69.72 ± 6.49%) than *T. gratilla* (43.37 ± 13.47%). Diversity analysis indicated that the gut digesta of sea urchin *T. gratilla* displayed a higher bacterial α-diversity compared with the other four species. PCoA showed that the four groups representing *D. setosum*, *D. savignyi*, *E. calamaris*, and *S. variolaris* were overlapping, but distant from the group representing *T. gratilla*. Predictive metagenomics performed by PICRUSt revealed that the abundances of genes involved in amino acid metabolism and metabolism of terpenoid and polyketide were higher in *T. gratilla*, while those involved in carbohydrate metabolism were higher in the other four sea urchin species. Therefore, our results indicated that the composition, diversity and predictive metabolic profiles of bacteria associated with the gut digesta of *T. gratilla* were significantly different from those of the other four sea urchin species in Luhuitou fringing reef.

## Introduction

Sea urchins play a dual role in the health and stability of coral reef ecosystems (Lawrence, 2013). Some sea urchin species, such as *Tripneustes gratilla* (Herring, 1972; Carreiro-Silva and McClanahan, 2001) and *Mierocyphus rousseau* (Herring, 1972; Rotjan and Lewis, 2008), play a positive role in coral growth and recruitment by feeding on algae, controlling their overgrowth. In 1983–1984, the sea urchin *Diadema antillarum* was nearly wiped out from the Caribbean coral reefs because of a disease outbreak, which resulted in a phase shift in the reef from coral to macroalgal dominance (Liddell and Ohlhorst, 1986; Lessios, 1988). The macroalgal cover increased by more than 50%, while the coral cover declined by an order of magnitude (Liddell and Ohlhorst, 1986; Hughes, 1994). Two decades later, the population of *D. antillarum* recovered at Discovery Bay, Jamaica, resulting in a reduction in the macroalgal cover and a concomitant increase in the densities of juvenile coral (Edmunds and Carpenter, 2001; Carpenter and Edmunds, 2010). On the south coast of Barbados, coral cover and the densities of juvenile corals on the leeward side of a seawall, which was frequently grazed by sea urchins, were significantly higher than that on the seaward side, which was not grazed by sea urchins (Macintyre et al., 2005). On the other hand, some sea urchin species, such as *Diadema setosum* (Carreiro-Silva and McClanahan, 2001; Qiu et al., 2014), *Echinometra mathaei* (Bronstein and Loya, 2014), and *Eucidaris thouarsii* (Herring, 1972; Glynn et al., 1979), negatively affect coral reefs by feeding on coral tissue and skeletons. In the Galapagos, sea urchins were a source of great concern as they led to the erosion of coral skeletons and limited coral growth (Herring, 1972). Sea urchin populations rapidly increased in a Kenyan reef lagoon, leading to the suppression of juvenile coral recruitment (O Leary et al., 2012). An outbreak of the sea urchin *D. setosum* led 23.7–90.3% of hard corals to either collapse or experience >25% tissue loss in Hong Kong (Dumont et al., 2013; Qiu et al., 2014). In many coral reefs, the rate of sea urchin bioerosion (3.6–9.1 kg CaCO_3_ m^−2^y^−1^) could equal or even exceed the rate of carbonate production (1–4 kg CaCO_3_ m^−2^y^−1^) (Bak, 1994). Thus, sea urchins can play a key role, either positive or negative, in maintaining coral reef ecology. However, most studies in this field thus far have mainly focused on the macroscopic ecology (e.g., feeding ecology and influence on coral and algae), with little research on the post-ingestive processes (e.g., food digestion and related processes) that are related to host macroscopic ecology.

Owing to their positive effects, some sea urchins have been employed in recent years to restore many degraded coral reefs (Stimson et al., 2007). For example, in Kāne’ohe Bay, the flourishing red algae *Kappaphycus* and *Eucheuma* block sunlight and smother the corals, resulting in vast destruction of coral reefs. In order to restore this ecosystem, three organizations have collaborated to rear the juvenile sea urchin *T. gratilla* and release the organisms in the bay in an attempt to clear the flourishing red algae for the proposed duration of 2009–2025^1^ (Stimson et al., 2007). In the Florida Keys, because the population of *D. antillarum* has yet to recover from the mass mortality event of 1983–1984 (mentioned above), the species has been captive-spawned and released into the wild to restore the coral reef (Sharp et al., 2017). Studies of the behavioral and morphological characteristics and genetic structure of sea urchins have been carried out to provide a scientific reference for development of a restoration plan (Francis-Floyd et al., 2015; Chandler et al., 2017; Sharp et al., 2017). However, little research has been conducted with a focus on their digestive physiology.

Recent advancements in culture-independent next-generation sequencing techniques have facilitated the study of gut-bacteria relationships and enhanced our understanding of the supportive role of gut bacteria in the growth, development, disease, digestive physiology, and environmental adaptability of the host (Seung Chul et al., 2011; Gomez et al., 2012; Nguyen, 2012; Guinane and Cotter, 2013). Thus far, studies about the gut bacterial community of sea urchin using culture-independent next-generation sequencing techniques have mainly focused on commercially important species such as *Lytechinus variegatus* (Lawrence et al., 2010; Hakim et al., 2015; Hakim et al., 2016), *Asterechinus elegans* (Becker et al., 2009) and *Paracentrotus lividus* (Meziti et al., 2007). These studies revealed that the bacterial community composition in *L. variegatus* was significantly different among the pharynx tissue, gut tissue, gut digesta, and fecal pellets (Hakim et al., 2015, 2016) and suggested that only the bacteria in the stomach and intestinal lumen were specifically symbiotic with the sea urchin (Meziti et al., 2007). Predictive metagenomics revealed that bacteria associated with the gut (spanning stomach and intestinal) tissue of *L. variegatus* had relatively high abundances of genes assigned to energy metabolism compared with those associated with their gut digesta, which had higher abundances assigned to carbohydrate, amino acid, and lipid metabolism (Hakim et al., 2016). However, the bacteria within gut digesta of ecologically important sea urchin species have been given relatively little attention.

In this study, five sea urchin species, including *Stomopneustes variolaris*, *Diadema setosum*, *Echinothrix calamaris*, *Diadema savignyi*, and *Tripneustes gratilla*, were collected from the Luhuitou fringing reef to analyze the gut contents and to investigate gut digesta bacterial community composition, predict the associated genetic functions, and identify the associated factor(s) shaping gut digesta bacterial composition and function. This study provides novel insight on bacteria within their gut digesta, and a foundation for the application of some sea urchin species in the restoration of coral reefs.

## Materials and Methods

### Study Site, Sample Collection, and Species Identification

**FIGURE 1:**
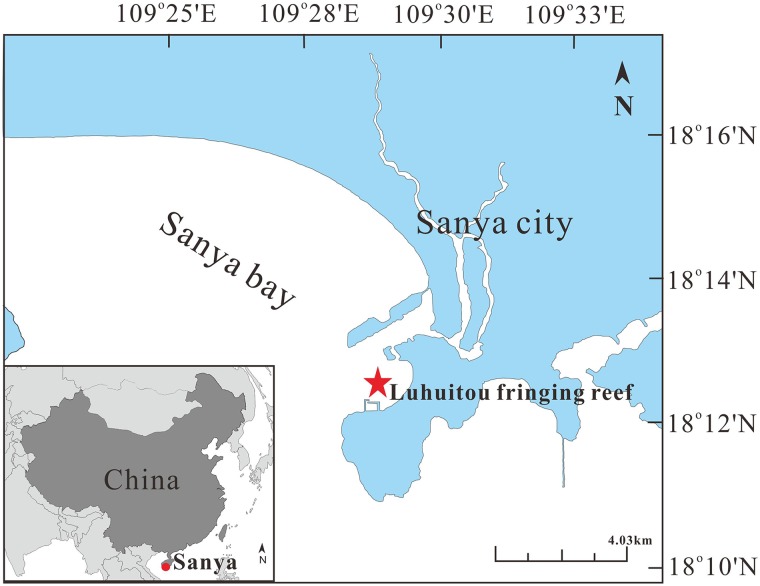
Map of study area. Sampling area represented by red circle and star.

All samples were collected in Luhuitou fringing reef (109°28′E, 18°13′N), located near Sanya City, Hainan Island, China (Yu, 2012). Luhuitou fringing reef is a typical fringing coral reef about 3 km long and 250–500 m wide, including reef flat and reef slope (Figure 1). The living corals are mainly distributed in areas with a water depth of less than 6 m (Zhao et al., 2012; Xu et al., 2017). The monthly mean sea surface temperature (SST) ranges from 23.1 to 29.8°C, with an annual SST of 27°C. The annual sea surface salinity (SSS) is 33.1%, ranging from 32.1 to 33.8% (Yu et al., 2010). The pH ranges from 7.5 to 8.4 (Cao et al., 2016). Because of increasing human activity, such as reef digging, overfishing, aquaculture, coastal construction, and diving tourism, the coral reefs have experienced a long-term decline, with the mean coral cover decreasing from 80–90% in 1962–1965 to just 9.52% in 2014 (Zhao et al., 2012; Jing et al., 2017).

Samples of sea urchins, which are generally found on coral or algae were collected at depths of 3–5 m by SCUBA between 10:00 am and 12:00 am on September 4, 2017. At least four replicates per species were collected within 2 h. At least one integral specimen of each sea urchin species was stored in 5% formaldehyde solution for species identification. Within 1 h of sampling, all remaining sea urchin samples were dissected using a sterile scalpel to obtain the digestive tract. The gut (spanning stomach and intestine) digesta was separated from the gut tissue and stored in 75% ethanol at −20°C until DNA extraction. All sea urchin specimens were identified according to their ecology and morphology. A light microscope (OLYMPUS, CX23) was employed to analyze the gut digesta samples.

### DNA Extraction and Illumina MiSeq Sequencing

The gut digesta was immersed in liquid nitrogen for 10 min and then freeze-dried to remove the residual ethanol. DNA from each sample was extracted with a ZR Soil Microbe DNA MiniPrep kit, according to the manufacturer’s instructions. After quality and purity evaluation, the V3–V4 region of the 16S rRNA was amplified from the extracted DNA using the bacteria-specific primer pair 338F (forward: 5′-ACTCCTACGGGAGGCAGCAG-3′) (Mori et al., 2014; Xu et al., 2016) and 806R (reverse: 5′-GGACTACHVGGGTWTCTAAT-3′) and the GeneAmp^®^9700 thermal cycler (Applied Biosystems, Foster City, CA, United States) (Sun et al., 2014). The fragments (421–460 bp in length) were purified using the AxyPrep DNA gel extraction kit (Axygen Bio-sciences, Union City, CA, United States) and QuantiFluor^TM^-ST fluorescence quantitative system (Promega, Madison, WI, United States). The purified amplicons were mixed at equimolar concentrations and then sequenced following a paired-end 250 bp × 2 strategy on an Illumina MiSeq platform operated by Majorbio Bio-Pharm Technology Co., Ltd., (Shanghai, China). The original reads were uploaded to the NCBI Sequence Read Archive database (Accession Number: PRJNA492364).

### Data Analysis

The raw sequences were trimmed and denoised by the software platform Trimmomatic to exclude the barcodes, adaptors, and low-quality reads (<20) (Bolger et al., 2014). The optimized sequences were aligned, and then the chimeric sequences were removed. Using the Ribosomal Database Project (RDP) as a reference, the remaining sequences were classified and clustered into operational taxonomic units (OTUs) based on 97% similarity (Edgar, 2010). Based on the clustered OTUs, a series of alpha diversity indices were calculated by Mothur (version v.1.30.1), including ACE, Chao, Shannon, and Simpson indices (Schloss et al., 2013), which were presented as the mean for each species. The taxonomic results were displayed at the phylum and genus levels by comparison with the SILVA database. The similarity of gut digesta bacterial composition among sea urchin species was analyzed with principal coordinates analysis (PCoA) and the analysis of similarity (ANOSIM) based on the unweighted UniFrac distances.

We used PICRUSt (Phylogenetic Investigation of Communities by Reconstruction of Unobserved States) to determine the predictive metagenomes of the gut digesta bacterial populations in each sea urchin sample (Langille et al., 2013). Closed-reference OTUs with 97% identity against the Greengenes database by Macqiime were selected for the PICRUSt analysis. The newly selected OTUs were normalized by the known/predicted 16S copy number abundance, and metagenomes were predicted using the “predict_metagenomes.py” module of PICRUSt. The predicted functional composition profiles were collapsed into hierarchical categories level 2 of Kyoto encyclopedia of genes and genomes (KEGG) database pathways. We also performed quality control steps for PICRUSt which gave the calculations of the reference genome coverage for each sea urchin sample presented as NSTI scores (Supplementary Table S10, supporting information). The relative abundances of functional categories of each sea urchin sample were calculated using Excel software.

The statistical analysis was performed by using STAMP v2.1.0 software. Homogeneity of variance was assessed on all data by using Levene’s test. We performed interspecific comparison of the abundance of three dominant bacterial phyla and the genus *Propionigenium* and the mean relative abundance of all metabolic pathway in KEGG level 2 by one-way ANOVA tests. Following significant ANOVA results, Tamhane’s T2 and SNK tests were used as *post hoc* multiple comparisons for further analysis of significance. Where variances were unequal, interspecific pair-wise comparisons of the numbers of OTUs, Shannon and Simpson indices and the total abundance of three dominant genera were tested using Welch’s *t*-test. If variance were equal, Student *t*-test were used. All results were presented in text as mean ± standard deviation. In all statistical analyses, the relatively conservative significance threshold of *p* < 0.01 was used.

## Results

### Host Sea Urchin Characterization

**FIGURE 2:**
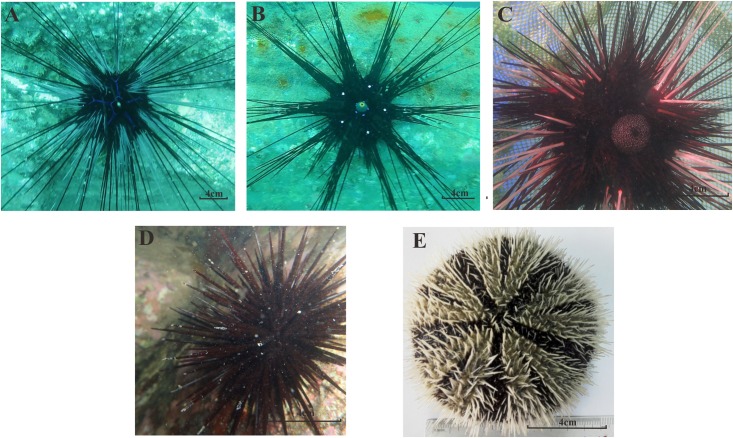
The morphological forms of all five sea urchin species in our study; **(A)** *D. savignyi*; **(B)** *D. setosum*; **(C)** *E. calamaris*; **(D)** *S. variolaris*; **(E)** *T. gratilla*.

A total of 40 sea urchin samples belonging to five species (*Stomopneustes variolaris*, *Diadema setosum*, *Echinothrix calamaris*, *Diadema savignyi*, and *Tripneustes gratilla*, Figure 2) and four genera were collected from the Luhuitou fringing reef. *T. gratilla*, *S. variolaris*, *D. setosum*, *E. calamaris*, and *D. savignyi* consisted of 20 individuals, 4 individuals, 7 individuals, 5 individuals, and 4 individuals, respectively (Table 1). The analysis indicated that the gut digesta in *T. gratilla* consisted almost entirely of macroalgae fragments, with no evidence of corals and their calcium carbonate skeleton, while the digesta of the other four sea urchin species were dominated by coral and their calcium carbonate skeleton, turf algae, filamentous algae, silt and calcareous algae.

**Table 1:** 16S rRNA gene sequencing data of Five Sea urchin.

Species	No. of replicates	No. of sequences		Good’s coverage (%)	Richness estimates			Diversity estimates	
					Observed OTUs	Chao	ACE	Shannon	Simpson
					
		Total	Mean	Mean	Mean	Mean	Mean	Mean	Mean
*S. variolaris*	4	243,096	60,774 ± 5353	99.8 ± 0.05%	368 ± 69	158 ± 26	163 ± 11	2.09 ± 0.26	0.26 ± 0.07
*D. setosum*	7	415,421	59,346 ± 6845	99.6 ± 0.23%	645 ± 365	212 ± 36	217 ± 28	2.16 ± 0.37	0.26 ± 0.07
*E. calamaris*	5	338,811	67,762 ± 2638	99.8 ± 0.06%	378 ± 79	172 ± 24	174 ± 19	2.06 ± 0.22	0.24 ± 0.04
*D. savignyi*	4	264,838	66,209 ± 8949	99.7 ± 1.10%	477 ± 67	204 ± 28	214 ± 45	2.15 ± 0.17	0.27 ± 0.04
*T. gratilla*	20	1,055,329	52,766 ± 5947	99.6 ± 0.08%	690 ± 78	256 ± 22	258 ± 22	3.06 ± 0.31	0.10 ± 0.04

### Alpha Diversity of Gut Digesta Bacteria in Sea Urchin

In total, 2,317,495 processed bacterial sequences were assigned to 1,708 OTUs at 97% similarity. The mean length of bacterial sequences was 440 bp. The mean number of reads was 56,131 in four to 20 replicates of each sea urchin species. Good’s coverage of gut digesta bacteria in each sea urchin sample was ≥99.6% of the diversity (Table 1), suggesting that our sequencing results represented the true condition of gut digesta bacteria in the sea urchin samples.

The number of OTUs did not differ significantly in pair-wise comparisons of the four sea urchin species *D. setosum*, *D. savignyi*, *E. calamaris*, and *S. variolaris* (*p* > 0.01, Supplementary Table S6, supporting information) but was significantly different in comparisons among the four sea urchin species with *T. gratilla* (*p* < 0.01, except for the comparisons of *D. setosum* – *T. gratilla*, where *p* = 0.016, Supplementary Table S6, supporting information). Furthermore, the number of OTUs was significantly higher in *T. gratilla* than in the four sea urchin species (Table 1 and Supplementary Table S6, supporting information). The mean number of OTUs was 690 ± 78 in *T. gratilla*, whereas it was 368 ± 69, 645 ± 365, 378 ± 79, and 477 ± 67 in *S. variolaris*, *D. setosum*, *E. calamaris*, and *D. savignyi*, respectively (Table 1 and Supplementary Table S1, supporting information).

The gut digesta bacterial alpha diversity indices of the sea urchin species, including Shannon and Simpson indices, are displayed in Table 1. The statistical analysis showed that the gut digesta bacterial alpha diversity indices (Shannon and Simpson indices) of the sea urchins did not differ significantly in pair-wise comparisons of the four sea urchin species *D. setosum*, *D. savignyi*, *E. calamaris*, and *S. variolaris* (*p* > 0.01, Supplementary Table S6, supporting information) but were significantly different in comparison of the four sea urchin species with *T. gratilla* (*p* < 0.01, except for the comparisons of *S. variolaris–T. gratilla* where *p* = 0.024, Supplementary Table S6, supporting information). In addition, compared with those of the other four sea urchin species, the Shannon indices were higher in *T. gratilla* (Table 1 and Supplementary Tables S1, S6, supporting information), whereas the Simpson indices were lower (Table 1 and Supplementary Tables S1, S6, supporting information), suggesting that the gut digesta bacterial alpha diversity in *T. gratilla* was higher than in the other four sea urchin species.

### Taxonomic Composition of Gut Digesta Bacteria

**FIGURE 3:**
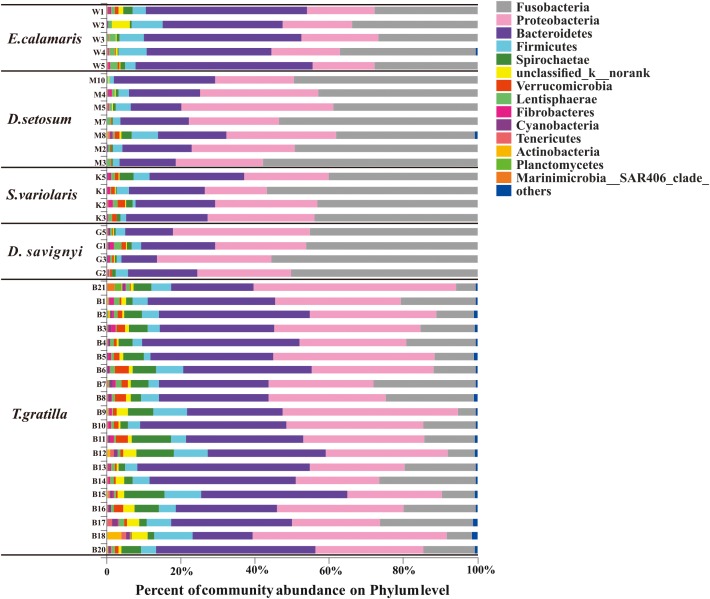
The gut bacterial community profiles of individual sea urchins at the phylum level. The horizontal axis represents the percentage of each phylum. Each bar represents the community of an individual sea urchin. Others denote those phyla whose abundances were less than 0.01%. W1–5 denote the five replicates of *E. calamaris*; M2–5, M7–8, and M10 denote the seven replicates of *D. setosum*; K1–3 and K5 denote the four replicates of *S. variolaris*; G1–3 and G5 denote the four replicates *D. savignyi*; B1–18 and B20–21 denote the 20 replicates *T. gratilla*.

Thirty-seven bacterial phyla were identified among the 40 sea urchin samples. Among them, 22 bacterial phyla were shared by the five sea urchin species, while 8 bacterial phyla were unique to *T. gratilla*. According to the mean abundance of each bacterial group, Fusobacteria (29.07 ± 16.39%), Proteobacteria (29.90 ± 9.07%), and Bacteroidetes (28.86 ± 10.30%) were predominant in all five sea urchin species (Supplementary Table S2, supporting information). However, the percentage of their abundance was significantly different among species (Fusobacteria *p* < 0.001; Proteobacteria *p* = 0.002; Bacteroidetes *p* < 0.001; Supplementary Table S7, supporting information). For example, Fusobacteria was the most abundant bacterial phylum in *D. setosum*, *D. savignyi*, and *S. variolaris*, accounting for 46.94 ± 7.51%, 49.15 ± 4.80%, and 45.84 ± 7.34%, respectively. Proteobacteria and Bacteroidetes represented the second and the third most abundant phyla, respectively, in the three sea urchin species. Although Fusobacteria (30.36 ± 4.48%) and Proteobacteria (18.60 ± 1.43%) were also highly abundant, Bacteroidetes (40.01 ± 6.68%) was the most abundant in *E. calamaris*. In *T. gratilla*, Bacteroidetes (33.60 ± 7.54%) and Proteobacteria (34.50 ± 8.99%) were the two most abundant bacterial phyla, while Fusobacteria accounted for 15.12 ± 7.08% (Figure 3 and Supplementary Table S2, supporting information).

**FIGURE 4:**
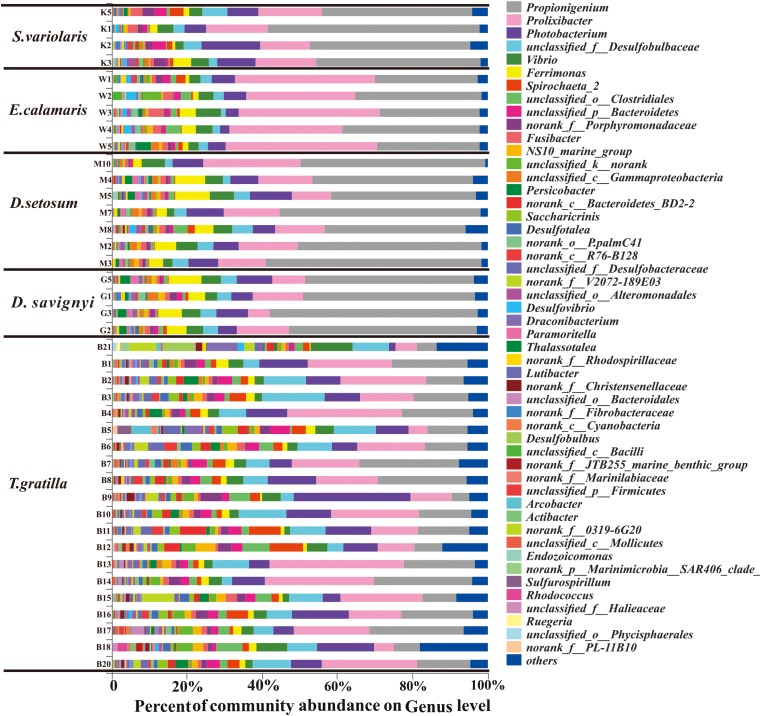
The gut bacterial community profiles of all individual sea urchins at the genus level. The horizontal axis represents the percentage of each genus. Each bar represents the community of an individual sea urchin. Others denote those genus whose abundances were less than 0.01%. Please refer to Figure 3 for sample identification.

In total, 438 genera were detected in the 40 sea urchin samples, with 420 genera in *T. gratilla*. Of these, 152 genera were shared by all five sea urchin species, while 3, 2, 3, and 111 genera were unique to *D. setosum, D. savignyi*, and *T. gratilla*, respectively. *Propionigenium*, *Prolixibacter*, and *Photobacterium* were the three most abundant bacterial genera in all five sea urchin species. The percentage of *Propionigenium* was significantly different among species (*p* < 0.001, Supplementary Table S7, supporting information). *Propionigenium* was the most abundant bacterial genus in the gut digesta of *D. setosum*, *D. savignyi*, and *S. variolaris*, accounting for 46.92 ± 7.53, 49.09 ± 4.79, and 45.81 ± 7.35%, respectively. *Prolixibacter* (34.78 ± 5.03%) and *Propionigenium* (30.35 ± 4.48%) were the two most abundant bacterial genera in *E. calamaris*. In *T. gratilla*, *Propionigenium* (15.04 ± 7.00%), *Prolixibacter* (18.05 ± 8.64%), and *Photobacterium* (10.28 ± 6.05%) showed similar abundance (Supplementary Table S3, supporting information). Other genera including *Vibrio* (1.39%–10.96%), *Ferrimonas* (0.31%–9.17%), *Spirochaeta_2* (0.07%–8.87%), *unclassified_o_clostridiales* (0.12%–7.31%), *unclassified_p_Bacteroidetes* (0.35%–5.37%), *NS10_marine-group* (0.14%–5.22%), *morank_f_Porphyromonadaceae* (0.22%–5.70%), *norank_c_Bacteroidetes_BD2-2* (0–7.04%), *Saccharicrinis* (0–4.34%), *unclassified_k_norank* (0.05%–4.65%), *Fusibacter* (0.14%–4.10%), *Persicobacter* (0.03%–4.06%), and *unclassified_c_Gammaproteobacteria* (0.11%–2.79%) were also abundant in the five sea urchin species (Supplementary Table S3, supporting information). The total abundance of the three dominant genera did not differ significantly in pair-wise comparisons of the four sea urchin species *D. setosum*, *D. savignyi*, *E. calamaris*, and *S. variolaris* (*p* > 0.01, Supplementary Table S8, supporting information) but was significantly different in comparison with the four sea urchin species with *T. gratilla* (*p* < 0.001, Supplementary Table S8, supporting information); the mean total abundance in the four sea urchin species (69.72 ± 6.49 %) was significantly higher than in *T. gratilla* (43.37 ± 13.47%, Figure 4 and Supplementary Table S8, supporting information).

### Sea Urchin Gut Digesta Bacterial Beta Diversity

**FIGURE 5:**
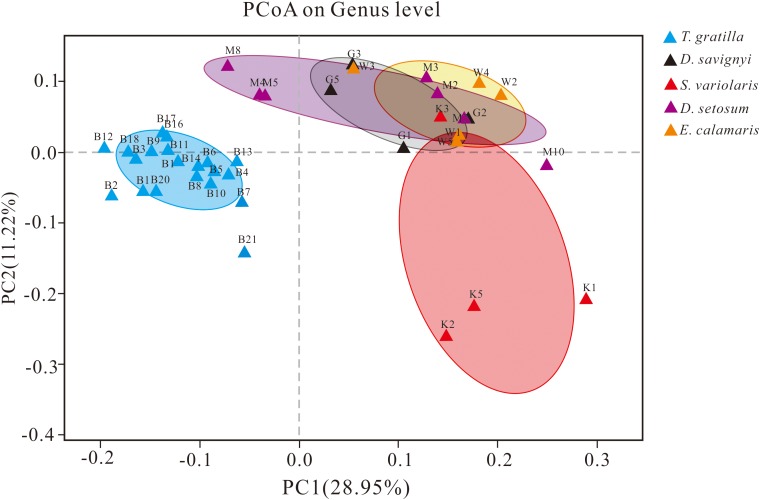
PCoA of the gut bacterial community of sea urchins at the genus level based on the subsampled unweighted UniFrac distance matrix at the genus level. PC1 and PC2 explained 28.95 and 11.22% of the total variance, respectively. For sample identification, please refer to Figure 3.

The similarity of gut digesta bacterial communities associated with the 40 sea urchin samples was evaluated using PCoA and ANOSIM based on the unweighted UniFrac distances at the genus level. PCoA, accounting for 28.95 and 11.22% of the total variance, showed that the four groups representing *D. setosum*, *D. savignyi*, *E. calamaris*, and *S. variolaris* were overlapping, but distant from the group representing *T. gratilla* (Figure 5).

ANOSIM revealed that the gut digesta bacterial composition did not differ significantly in pair-wise comparisons of the four sea urchin species *D. setosum*, *D. savignyi*, *E. calamaris*, and *S. variolaris* (*p* > 0.01, Table 2) but was significantly different in comparison of the four sea urchin species with *T. gratilla* (*p* = 0.001, Table 2).

**Table 2:** ANOSIM of unweighted UniFrac distance among the bacterial composition associated with the gut digesta of five sea urchin species at the genus level.

Interaction	*r*	*P*	Interaction	*r*	*P*
*D. setosum*–*D. savignyi*	−0.11	0.735	*E. calamaris*–*S. variolaris*	0.54	0.016
*D. setosum*–*E. calamaris*	0.15	0.013	*E. calamaris*–*T. gratilla*	0.92	0.001
*D. setosum*–*S. variolaris*	0.54	0.018	*D. savignyi*–*T. gratilla*	0.77	0.001
*D. savignyi*–*E. calamaris*	0.28	0.105	*D. setosum–T. gratilla*	0.63	0.001
*D. savignyi*–*S. variolaris*	0.55	0.054	*S. variolaris– T. gratilla*	0.96	0.001

### Gut Digesta Bacterial Gene Functions of Sea Urchin

**FIGURE 6:**
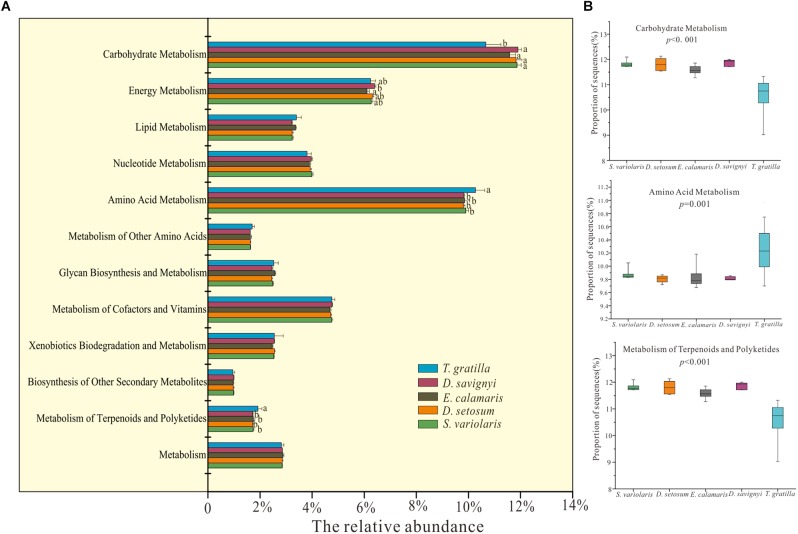
PICRUSt (V1.0.0) analysis of predicted metagenomes using the 16S rRNA gene data of gut digesta bacteria of *D. savignyi*, *D. setosum*, *E. calamaris*, *S. variolaris*, and *T. gratilla*. **(A)** The mean relative abundance of all metabolic pathways in KEGG level 2 in the five sea urchin species. The error bars denote the standard deviation. **(B)** Box plots of the KEGG level 2 categories of carbohydrate metabolism, amino acid metabolism and metabolism of terpenoids and polyketides analyzed by STAMP v2.1.0 software. The *p*-values of each KEGG level 2 analysis are displayed in the box plot.

Metagenomics predicted by PICRUSt revealed 41 functional categories in level 2 of KEGG. The statistical analysis showed that the mean relative abundances of most of functional categories in level 2 of KEGG were significantly different among all five sea urchin species (Supplementary Table S4, supporting information). Metabolism accounted for the most abundant genes in the gut digesta bacteria of sea urchin, with *D. setosum* 52.01 ± 0.43%, *D. savignyi* 51.34 ± 0.2%, *S. varilaris* 52.23 ± 0.23%, *E. calamaris* 51.74 ± 0.53%, and *T. gratilla* 51.62 ± 1.16%, respectively (Supplementary Table S9, supporting information). In the metabolic pathway maps, the mean relative abundances of genes involved amino acid metabolism, metabolism of terpenoid and polyketide and carbohydrate metabolism did not differ significantly in pair-wise comparisons of the four sea urchin species *D. setosum*, *D. savignyi*, *E. calamaris*, and *S. variolaris* (*p* > 0.01, Figure 6 and Supplementary Table S9, supporting information) but was significantly different in comparisons between the four sea urchin species and *T. gratilla* (*p* < 0.01, Figure 6 and Supplementary Table S9, supporting information). The mean relative abundances of genes involved with amino acid metabolism and terpenoid and polyketide metabolism were significantly higher in *T. gratilla* than in the four sea urchin species (Figure 6 and Supplementary Table S9, supporting information). However, the mean relative abundances of genes associated with carbohydrate metabolisms were higher in the four sea urchin species than in *T. gratilla* (Figure 6 and Supplementary Table S9, supporting information). The mean relative abundance of genes from many KEGG level 3 subcategories also differed significantly between the two groups (Supplementary Table S5, supporting information).

## Discussion

### Gut Digesta Bacterial Composition of Sea Urchins in Luhuitou Fringing Reefs

In our study, more than three replicates of each sea urchin species together with high-throughput sequencing techniques contributed to a reliable taxonomic identification of bacterial communities and the predictive metagenomics in the gut digesta of sea urchins. The results revealed that the gut digesta of the five examined sea urchin species from Luhuitou fringing reefs consisted mainly of bacteria belonging to three bacterial phyla: Fusobacteria, Proteobacteria, and Bacteroidetes. The gut digesta bacterial community composition of these sea urchins was different from those reported in sea urchins growing in other ecosystems. For example, the gut digesta bacteria of the sea urchin *L. variegatus* growing in a natural habitat (Hakim et al., 2016), laboratory (Hakim et al., 2015), or aquaculture pool (Lawrence et al., 2010) were mainly dominated by Proteobacteria. Members of the phylum Fusobacteria dominated the gut digesta of all five sea urchin species in our study; however, based on previous reports, they are seldom found to dominate the gut digesta bacterial community of sea urchins. Zha et al. (2018) reported that Fusobacteria could be used as indicators of a host experiencing stress from food resource limitation and heavy predation. Furthermore, many bacterial species of the phylum Fusobacteria have been associated with infections caused by host psychological stress (Bennett and Eley, 1993). Therefore, we inferred that sea urchins in Luhuitou fringing reef might be experiencing some sort of stress. There are two likely sources of stress faced by the sea urchins. First, Luhuitou fringing reef has experienced a long-term decline owing to anthropogenic activities and environmental degradation, leading to a decrease in the mean coral cover from 80–90% in 1962–1965 (Zhao et al., 2012) to just 9.52% in 2014 (Jing et al., 2017). This suggests that the living conditions of organisms were greatly affected, accounting for some of the stress faced by the sea urchins inhabiting this niche. Second, the shelters available to sea urchins for escaping predation were lost due to coral reef loss, thus increasing their stress from predators. Members of the phylum Bacteroidetes also dominated the gut digesta of all five sea urchin species collected from Luhuitou fringing reef. These bacteria are considered specialists in the degradation of high-molecular-weight organic matter, i.e., proteins and carbohydrates (Thomas et al., 2011). Complex polysaccharides are resistant to the action of digestive enzymes (Thomas et al., 2011). The members of the phylum Bacteroidetes are believed to complement eukaryotic genomes with degradation enzymes targeting resistant polymers such as plant cell wall compounds (Thomas et al., 2011). The recent sequencing of Bacteroidetes revealed numerous carbohydrate-active enzymes that can degrade a broad spectrum of substrates of plant, algal, and animal origin (Thomas et al., 2011).

In the gut digesta of five sea urchin species, the predominant bacterial genera were *Propionigenium*, *Prolixibacter*, and *Photobacterium*. The genus *Propionigenium* of the phylum Fusobacteria currently comprises two known species, both obligate anaerobes: *P. modestis* and *P. maris* (Schink, 2006). These are known to translate succinate into propionate in enrichment culture (Schink, 2006). Our results showed that *Propionigenium* accounted for almost half of the gut digesta bacteria, suggesting that propionate may be one of the main microbial metabolites in the gut digesta of sea urchins in Luhuitou fringing reef. Propionate was found to be a health-promoting microbial metabolite, and its production may be promoted when organisms are in unfavorable environments (Hosseini et al., 2011). These results suggest that the sea urchins in our study may have experienced stress. The genus *Prolixibacter* of the phylum Bacteroidetes comprises facultative anaerobes, and some of these species can ferment sugars through the acid fermentation pathway (Holmes et al., 2007). *Photobacterium* of phylum Proteobacteria was also abundant in the sea urchin *P. lividus* (Meziti et al., 2007). This bacterial genus consists of 16 known species, some of which are closely linked with lipolytic activity (Yoon et al., 2005; Schlosser et al., 2015). Another abundant genus, *Vibrio*, represented the dominant bacteria in the gut digesta of the sea urchin *L. variegatus* (Lawrence et al., 2010; Hakim et al., 2015, 2016). Members of *Vibrio* isolated from the gut of sea urchin *Strongylocentrotus nudus* produced amylase, gelatinase, chitinase, and fucosidanase (Beleneva and Kukhlevskii, 2010), suggesting that they may participate in the host’s food digestion process.

### Factors Driving the Gut Digesta Bacterial Alpha and Beta Diversity of Sea Urchin

According to our gut digesta analysis, *T. gratilla* primarily feed on macroalgae, while the other four sea urchin species, *D. setosum*, *D. savignyi*, *E. calamaris*, and *S. variolaris*, mainly feed on coral skeletons, filamentous, turf algae, and other animals. The results of our gut digesta analysis were similar to those in previous studies (Herring, 1972). For example, Herring reported that seagrasses were dominant within the stomach content of *T. gratilla*, while the stomach contents in both *E. calamaris*, *D. setosum* and *S. variolaris* were dominated by algae, coral, silt and calcareous algae (Herring, 1972). Carreiro-Silva and McClanahan (2001) reported that *D. setosum* and *D. savignyi*, as scraper, erode coral skeletons, but *T. gratilla*, as a browser, do not. Furthermore, PCoA and ANOSIM showed that the gut digesta bacterial composition did not differ significantly in pair-wise comparisons of the four sea urchin species but was significantly different in comparisons of the four sea urchin species with *T. gratilla*. Many previous studies have showed that diet was one of the key factors shaping the gut bacterial composition of many wild animals (Miyake et al., 2015). For example, the gut bacterial communities among carnivorous, omnivorous, and herbivorous animals were found to be significantly different (Ley et al., 2008; Delsuc et al., 2014). In fact, seasonal variations in the gut bacterial community are mainly driven by seasonal shifts in diet composition (Carey et al., 2013; Maurice et al., 2015; Xue et al., 2015; Ren et al., 2017). Therefore, the different diet among *T. gratilla* and the other four sea urchin species might be also one of the forces shaping the difference of their gut digesta bacteria community in the Luhuitou fringing reef.

Our results also indicated that *T. gratilla* displayed a higher gut digesta bacterial alpha diversity than the other four sea urchin species. According to our gut digesta analysis supported previous studies, with *T. gratilla* feeding primarily on macroalgae, while the other four sea urchin species ingested a greater diversity of food, including smaller filamentous and turf algae, coral and coral skeletons and other animals (Herring, 1972; Birkeland, 1989; Stimson et al., 2007). Thus, the gut digesta bacterial diversity of sea urchins in our study did not display a positive relationship with the host’s dietary diversity. This result reinforces that other factors besides diet diversity may contribute to shape the gut digesta bacterial alpha diversity of sea urchin. Previous studies of pika and freshwater fish also reported a similar phenomenon (Bolnick et al., 2014; Li et al., 2016, 2018). It is reported that host diet and phylogeny both influence bacterial alpha diversity (Ley et al., 2008). Sea urchin phylogeny may also impact the alpha diversity of gut digesta bacterial communities in Luhuitou finging reef.

### Functional Categories of Gut Digesta Bacteria of Five Sea Urchin Species

Sea urchins lack certain digestive enzymes in the gut (Lawrence et al., 2010), and their gut bacteria are considered to play an important role in the digestion and metabolism of certain food substances and in the synthesis of key biomolecules (Becker et al., 2009; Scott et al., 2013). Consistent with this, the results of predictive metagenomics indicated that metabolism accounted for the most abundant genes in the gut digesta bacteria of sea urchin. Moreover, the average relative abundance of functional categories corresponding to carbohydrate, amino acid, lipid, and energy metabolism in our study was similar to that in the gut digesta of sea urchin *L. variegatus* from its natural habitat (Hakim et al., 2016).

Some of the pathway maps in the KEGG level 2 functional modules differed significantly between the four sea urchin species (*D. setosum*, *D. savignyi*, *E. calamaris*, and *S. variolaris*) and *T. gratilla*, which might be closely related to the animal-based diet of *D. setosum*, *D. savignyi*, *E. calamaris*, and *S. variolaris*. For example, the mean relative abundance of amino acid metabolism was higher in the four sea urchin species than in *T. gratilla* (Figure 6). The animal-based diet might consist of more amino acids that can be directly utilized by the sea urchins. Therefore, chemical reactions associated with the metabolism, biosynthesis, and degradation of various necessary amino acids were less prevalent in the four sea urchin species owing to the animal-based diet.

## Conclusion

In this study, we found that the gut digesta bacterial composition of five sea urchin species was remarkably different from those reported for sea urchins in previous studies. The data provided evidence that sea urchins in Luhuitou fringing reef might experience stress. The composition, diversity, and functional categories of gut digesta bacteria were significantly different among *T. gratilla* and the other four sea urchin species *D. setosum*, *D. savignyi*, *E. calamaris*, and *S. variolaris*.

## Author Contributions

KY, JL, and QY conceived the research. YW, BH, XH, BC, and ZQ provided the materials. QY conducted all experiments. QY wrote the manuscript. All authors edited and approved the manuscript.

## Conflict of Interest Statement

The authors declare that the research was conducted in the absence of any commercial or financial relationships that could be construed as a potential conflict of interest.

## Footnotes

1 https://www.fpir.noaa.gov/stories/02012017_sea_urchins_are_saving_kaneohebay.html

## Supplementary Material

TABLE S1 The specific value of sobs, Shannon, Simpson, ace, Chao, and coverage of each sample.

TABLE S2 The percent of all individual sea urchins at the phylum level.

TABLE S3 The percent of all individual sea urchins at the genus level.

TABLE S4 The mean relative abundance of each predicted functional category in KEGG pathway (level 2).

TABLE S5 The mean relative abundance of each predicted functional category in KEGG pathway (level 3).

TABLE S6 Result of *t*-test in the pair-wise comparisons of the numbers of OTUs and alpha-diversity values in bacterial communities characterized within the gut digesta of five sea urchin species.

TABLE S7 Results of ANOVA analysis comparing the proportion of three dominant phyla (Fusobacteria, Proteobacteria, and Bacteroidetes) and the genus Propionigenium among the five sea urchin species.

TABLE S8 The statistical result of a pair-wise comparisons using the total percent of three dominant genus (Propionigenium, Prolixibacter, and Photobacterium) of five sea urchin species.

TABLE S9 The original data of Figure 6.

TABLE S10 The specific value of NSTI scores of each sample.
