# Supplementary material for: The Composition, Diversity and Predictive Metabolic Profiles of Bacteria Associated With the Gut Digesta of Five Sea Urchins in Luhuitou Fringing Reef (Northern South China Sea)
Source: Front Microbiol. 2019 May 28;10:1168. doi: 10.3389/fmicb.2019.01168 (PMC6546719; doi:10.3389/fmicb.2019.01168)
Supplement: TABLE S6 — Result of t-test in the pair-wise comparisons of the numbers of OTUs and alpha-diversity values in bacterial communities characterized within the gut digesta of five sea urchin species. [file Table_6.doc]

**Supplementary Material**

**Table S6** Result of t-test in the pair-wise comparisons of the numbers of OTUs and alpha-diversity values in bacterial communities characterized within the gut digesta of five sea urchin species

|  | **the numbers of OTUs** | **SHANNON** | **SIMPSON** |
| --- | --- | --- | --- |
| **Interaction** | **Pvalue** | **Pvalue** | **Pvalue** |
| *D. setosum*–*D. savignyi* | 0.9677 | 0.5654 | 0.5338 |
| *D. setosum*–*E. calamaris* | 0.2353 | 0.9157 | 0.5315 |
| *D. setosum*–*S. variolaris* | 0.1919 | 0.7327 | 0.6451 |
| *D. savignyi*–*E. calamaris* | 0.08293 | 0.3769 | 0.9532 |
| *D. savignyi*–*S. variolaris* | 0.06478 | 0.307 | 0.3344 |
| *E. calamaris*–*S. variolaris* | 0.8395 | 0.566 | 0.3355 |
| *E. calamaris*–*T. gratilla* | 0.000194 | 5.84E-09 | 0.00017 |
| *D. savignyi*–*T. gratilla* | 0.002625 | 1.44E-05 | 0.005005 |
| *D. setosum–T. gratilla* | 0.01563 | 0.000561 | 0.003821 |
| *S. variolaris–T. gratilla* | 0.000579 | 0.001829 | 0.02447 |

Table S7 Results of ANOVA analysis comparing the proportion of three dominant phyla (Fusobacteria, Proteobacteria and Bacteroidetes) and the genus Propionigenium among the five sea urchin species

| **Bcteria** | ***P*** | **Bcteria** | ***P*** |
| --- | --- | --- | --- |
| Fusobacteria | ＜0.001 | Bacteroidetes | ＜0.001 |
| Proteobacteria | 0.002 | *Propionigenium* | ＜0.001 |

Table S8 the statistical result of a pair-wise comparisons using the total percent of three dominant genus (Propionigenium, Prolixibacter and Photobacterium) of five sea urchin species

| **Interaction** | **Pvalue** | **Interaction** | **Pvalue** |
| --- | --- | --- | --- |
| *D. setosum*–*D. savignyi* | 0.398 | *E. calamaris*–*S. variolaris* | 0.532 |
| *D. setosum*–*E. calamaris* | 0.681 | *E. calamaris*–*T. gratilla* | < 0.001 |
| *D. setosum*–*S. variolaris* | 0.86 | *D. savignyi*–*T. gratilla* | < 0.001 |
| *D. savignyi*–*E. calamaris* | 0.123 | *D. setosum–T. gratilla* | < 0.001 |
| *D. savignyi*–*S. variolaris* | 0.188 | *S. variolaris–T. gratilla* | 0.001 |

Table S9 the original data of fig 6

|  | ***S. variolaris*** | | ***D. setosum*** | | ***E. calamaris*** | | ***D. savignyi*** | | ***T. gratilla*** | |  |
| --- | --- | --- | --- | --- | --- | --- | --- | --- | --- | --- | --- |
| **pathway** | **mean** | **Standard Deviation** | **mean** | **Standard Deviation** | **mean** | **Standard Deviation** | **mean** | **Standard Deviation** | **mean** | **Standard Deviation** | **Pvalue** |
| Metabolism | 2.846398 | 0.0151798 | 2.852036 | 0.0301243 | 2.875044 | 0.0388559 | 2.855742 | 0.0063311 | 2.818552 | 0.0989376 | 0.705 |
| Metabolism of Terpenoids and Polyketides | 1.734704 | 0.0329542 | 1.717847 | 0.0224395 | 1.738625 | 0.0645376 | 1.721376 | 0.0122421 | 1.924873 | 0.1455536 | 0.000 |
| Biosynthesis of Other Secondary Metabolites | 0.990122 | 0.0118157 | 0.977414 | 0.0248929 | 0.971364 | 0.0240347 | 0.993395 | 0.0154593 | 0.960693 | 0.0665564 | 0.691 |
| Xenobiotics Biodegradation and Metabolism | 2.528345 | 0.0191089 | 2.556768 | 0.0224752 | 2.471718 | 0.0309159 | 2.542970 | 0.0126697 | 2.547862 | 0.3442155 | 0.981 |
| Metabolism of Cofactors and Vitamins | 4.754218 | 0.0351348 | 4.716632 | 0.0337596 | 4.691644 | 0.0340590 | 4.763570 | 0.0205492 | 4.755198 | 0.1115370 | 0.533 |
| Glycan Biosynthesis and Metabolism | 2.489565 | 0.0203259 | 2.450237 | 0.0333695 | 2.569167 | 0.0349703 | 2.459739 | 0.0364618 | 2.533164 | 0.1671127 | 0.401 |
| Metabolism of Other Amino Acids | 1.628085 | 0.0108651 | 1.629455 | 0.0111095 | 1.632485 | 0.0385624 | 1.624214 | 0.0075971 | 1.698742 | 0.0878629 | 0.004 |
| Amino Acid Metabolism | 9.902234 | 0.1006089 | 9.808923 | 0.0528418 | 9.851851 | 0.1997838 | 9.821369 | 0.0315458 | 10.27527 | 0.3478293 | 0.001 |
| Nucleotide Metabolism | 3.991982 | 0.0455349 | 3.928145 | 0.0536768 | 3.892257 | 0.0524000 | 3.967494 | 0.0378710 | 3.805243 | 0.1597852 | 0.017 |
| Lipid Metabolism | 3.231871 | 0.0393656 | 3.233665 | 0.0358604 | 3.366686 | 0.0355952 | 3.213584 | 0.0239258 | 3.392928 | 0.1976056 | 0.041 |
| Energy Metabolism | 6.270291 | 0.0599120 | 6.310634 | 0.0554824 | 6.109934 | 0.0836518 | 6.397132 | 0.0496919 | 6.245842 | 0.1907738 | 0.066 |
| Carbohydrate Metabolism | 11.864697 | 0.1654331 | 11.826582 | 0.2250522 | 11.578238 | 0.2251273 | 11.905767 | 0.1282852 | 10.666369 | 0.5794105 | <0.001 |
